# Daily Micro Moments (DMMs): A Feasibility Framework for Integrating Lifestyle Medicine to Enhance Physician Well-Being and Reduce Burnout

**DOI:** 10.7759/cureus.96002

**Published:** 2025-11-03

**Authors:** Sumera Bukhari

**Affiliations:** 1 Internal Medicine/Hospital Medicine/Lifestyle Medicine, Cambridge Health Alliance, Harvard Medical School, Boston, USA

**Keywords:** framework, lifestyle intervention, logbook, mindfulness-based therapy, s: physician burnout, technical report, wellness and resilience

## Abstract

Physician well-being is foundational to safe, compassionate, and sustainable care, yet remains difficult to sustain in the high-demand clinical environment. Although awareness of clinician stress and fatigue is growing, many wellness initiatives are resource-intensive, require protected time, or lack sustainability when physicians face competing demands. Early-career clinicians and trainees are particularly vulnerable, often balancing academic and clinical responsibilities with limited opportunities for recovery.

This technical report describes the design and pilot implementation of a simple logbook-based framework that integrates brief lifestyle “micro-practices” into daily routines as a resilience-building strategy. The framework was developed using quality-improvement principles, specifically Plan-Do-Study-Act (PDSA) cycles, emphasizing feasibility, rapid feedback, and sustainability. It comprised three elements: (1) short lifestyle micro-practices lasting three to five minutes (e.g., mindful breathing, gratitude journaling, reflective pauses); (2) a structured logbook serving as both an accountability tool and a data-capture instrument; and (3) balancing measures to ensure participation did not disrupt academic or clinical duties.

The framework was piloted over a three-week period by a physician concurrently enrolled in full-time graduate coursework while providing inpatient care on weekends - a high-demand context to test feasibility. Adherence to daily practices exceeded 80%. The Mini-Z single-item burnout score improved by one level, and perceived-stress ratings declined from 8 → 6 → 5 across three weeks. The logbook reinforced engagement by visualizing progress, while balancing measures confirmed no interference with coursework or clinical responsibilities.

This pilot demonstrates that integrating structured lifestyle micro-practices into physician routines is feasible and potentially beneficial, even under dual academic and clinical workloads. By embedding well-being within natural transitions rather than separate sessions, the framework aligns with established quality-improvement principles and supports sustainable resilience without requiring additional time. Future studies should evaluate this approach in larger cohorts using validated well-being and resilience instruments to determine broader impact and scalability.

## Introduction

Workforce well-being has become an urgent concern in modern medicine, as clinicians, trainees, and students navigate high workloads, administrative demands, and limited opportunities for recovery during the workday. Burnout remains a widely recognized problem with significant repercussions for physicians, patients, and health systems [1,2]. Numerous wellness interventions have been introduced, ranging from structured institutional programs to mindfulness training and organizational reforms [3]. While many of these approaches demonstrate benefit, they often require protected time or significant resources, limiting their feasibility in already time-pressured environments [4]. Consequently, a persistent gap remains between the intent of wellness initiatives and their practical integration into daily clinical life. Scholars have emphasized that interventions must be not only effective but also feasible and sustainable in real-world settings [3]. Building on this premise, the present report focuses on feasibility within the daily routines of clinicians, trainees, and students, proposing that well-being strategies must integrate seamlessly into existing workflows rather than compete with professional responsibilities.

To address this gap, we introduce the concept of Daily Micro Moments (DMMs) - brief, intentional pauses lasting only a few minutes, embedded within the natural flow of the day. These may occur during transitions between tasks, brief breaks between patient encounters, or pauses between academic sessions. Unlike casual diversions, DMMs rely on deliberate awareness rather than disengagement. Their restorative effect arises from purposeful attention within ordinary transitions. DMMs are designed to promote resilience and well-being without requiring additional time, making them highly adaptable for fast-paced, high-demand environments.

Although developed for clinical practice, the DMM framework’s simplicity and flexibility make it broadly applicable across settings where time for self-care is limited. Rather than prescribing specific activities, it provides a structure adaptable to personal preferences, professional roles, and training levels. By embedding restoration into the natural rhythm of work, DMMs reframe well-being from an additional task into an integrated practice. This model emphasizes feasibility, sustainability, and presence, offering a pragmatic approach to maintaining focus and resilience amid the everyday realities of medical practice.

## Technical report

This project was designed as a practical, real-world pilot to test whether very short wellness activities could be integrated into the daily routine of a physician balancing multiple responsibilities. Rather than adding new scheduled time, the goal was to weave intentional pauses directly into the natural flow of work and study. The design was guided by quality-improvement (QI) principles, particularly the use of Plan-Do-Study-Act (PDSA) cycles to conduct small tests of change and balancing measures to monitor unintended effects [5].

Framework

The intervention was structured around three QI-aligned elements derived from the Institute for Healthcare Improvement’s Science of Improvement framework [5].

Daily Micro Moments (DMMs)

Brief, intentional pauses lasting three to five minutes that could be performed anywhere. The physician selected from a predefined menu of micro-practices such as mindful breathing, brief gratitude reflection, or centering pauses between activities. These moments were designed to occur during existing transitions-before rounds, between classes, or after documentation-rather than as separate scheduled sessions.

Structured Logbook

A daily record used to track adherence, duration, timing reliability, and brief reflections on stress and focus. The logbook functioned as both a research and implementation tool, enabling systematic data collection for process and outcome evaluation. Its purpose was to assess feasibility, adherence, and timing patterns during the initial PDSA cycles. In future stages - once the framework is scaled and validated across broader cohorts - the structured log may no longer be required, as established feasibility data could guide natural integration without added documentation burden.

Balancing Measures

Weekly indicators assessing potential unintended effects, including perceived burden (1-5 scale) and time displacement from academic or clinical duties. These were included to ensure the intervention did not interfere with professional responsibilities. Once feasibility and non-disruption are empirically established, such measures could evolve into periodic qualitative check-ins rather than continuous data collection.

Together, these components created a structured yet flexible framework for embedding intentional, measurable Daily Micro Moments into the workday while maintaining alignment with established QI principles. Examples of DMMs incorporated into the pilot and proposed for future implementation are presented in Table 1.

**Table 1:** Examples of Daily Micro Moments (DMMs) Examples in this table are based on practices piloted in this project and proposed as part of the Daily Micro Moments (DMMs) framework. These are intended to illustrate how DMMs can be embedded into natural transitions without requiring a separate scheduled time

Category	Example	Duration	Integration point
Mindful breathing	Three slow, intentional breaths to reset	1–2 minutes	Transition between patients or tasks
Gratitude journaling	Write or silently note one thing you are grateful for	2–3 minutes	Before/after class or rounds
Reflective pause	Think of one meaningful interaction from the day	2 minutes	End of shift or study session
Grounding exercise	Notice five things you can see, four you can feel, three you can hear	3 minutes	Waiting periods, documentation breaks
Silent reset	Sit quietly, close your eyes, and take three breaths	1 minute	Mid-shift or between academic tasks
Color grounding	Identify three objects of the same color around you	1–2 minutes	Any transition moment
Micro-meditation	Focus on one sound in the environment	1 minute	Short pause before next task
Mini-movement	Stretch shoulders/neck or stand briefly	1–2 minutes	Between meetings or while studying
Intentional hydration	Drink water slowly with mindful awareness	1 minute	Before starting the next patient or class
Positive affirmation	Quietly repeat a calming/empowering phrase	1 minute	Before rounds, exams, or presentations

Context

The DMM framework was piloted over a three-week period (July 21-August 14, 2025) by a hospitalist concurrently enrolled in full-time graduate coursework while providing inpatient care on weekends. This dual workload created a high-demand environment that served as a practical “stress test” for feasibility. The pilot used a structured measurement plan informed by QI methods, incorporating both process measures and validated outcome data. The Mini-Z single-item burnout score (0-4 scale) was collected at baseline and completion to quantify changes in self-reported burnout.

Although this initial feasibility project involved a single participant, the framework was intentionally designed to be flexible and adaptable, supporting translation to diverse professional contexts. The model applies not only to physicians but also to trainees, residents, and students managing similarly fragmented schedules and high cognitive demands.

Implementation and adherence

Three DMMs were selected at the start of the pilot, guided by QI principles from the Institute for Healthcare Improvement (IHI) Model for Improvement. The selected DMMs included a brief three to five-minute mindfulness or grounding pause, daily logbook tracking of practice frequency and duration, and a short weekly reflection. The physician aimed to complete one DMM each day and documented adherence and reflections immediately afterward in the logbook.

Implementation was monitored through two iterative PDSA cycles to test and refine timing and consistency:

Cycle 1: Introduced a nightly reminder cue to improve consistency, increasing completion from three to four to six of seven days.

Cycle 2: Added a three-minute pause before transitions between academic and clinical tasks, completed in five of seven planned pauses, with lower stress ratings and improved focus.

Details of both PDSA cycles are summarized in Table 2.

**Table 2:** Summary of Plan-Do-Study-Act (PDSA) cycles conducted to refine timing and consistency of Daily Micro Moments (DMMs) Table 2 summarizes the two Plan-Do-Study-Act (PDSA) cycles used to refine the implementation of Daily Micro Moments (DMMs). Both cycles demonstrated improved adherence and reduced stress without disrupting clinical or academic responsibilities

Cycle	Test of change	Plan	Do	Study/results	Act/next step
1	Nightly reminder cue to improve consistency	Introduce an 8 PM phone reminder to prompt completion of a Daily Micro Moment before study tasks.	Implemented for one week; baseline completion three to four days/week	Adherence improved to six of seven days; minor timing conflicts with classwork	Adopt a reminder and adjust timing to 9 PM for a better fit
2	Three-minute pause before task transitions	Add a brief three-minute grounding pause after class or before the inpatient shift	Practiced five of seven planned pauses	Stress ratings decreased (3.5 → 2.5) and focus ratings increased (3 → 4)	Adopt practice; simplify to one to two minutes on high-demand days

Across the three-week period, adherence to daily DMMs exceeded 80% (20 of 25 days). Of note, 92% of sessions stayed within the three to five-minute duration target, and approximately 80% occurred within the intended evening or transition windows. Missed sessions were limited to post-overnight shifts, when fatigue prevented participation.

**Figure 1:**
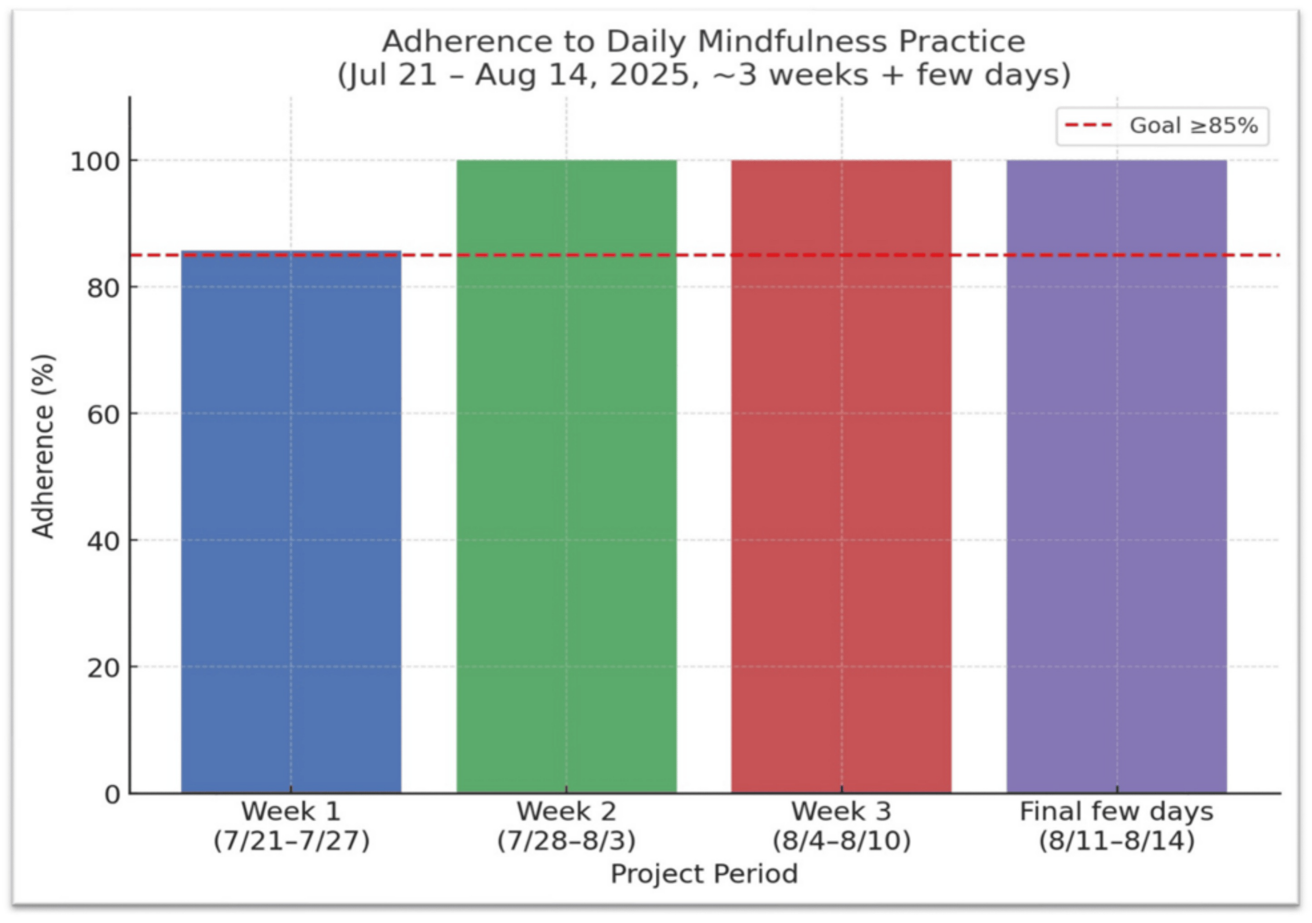
Weekly adherence to Daily Micro Moments (DMMs) Weekly adherence to Daily Micro Moments (DMMs) over the three-week pilot period (July 21-August 14, 2025). Adherence remained above 80% each week, meeting the project’s target threshold of ≥ 85%. These findings demonstrate the feasibility of sustaining short, intentional wellness pauses during routine clinical and academic work

**Figure 2:**
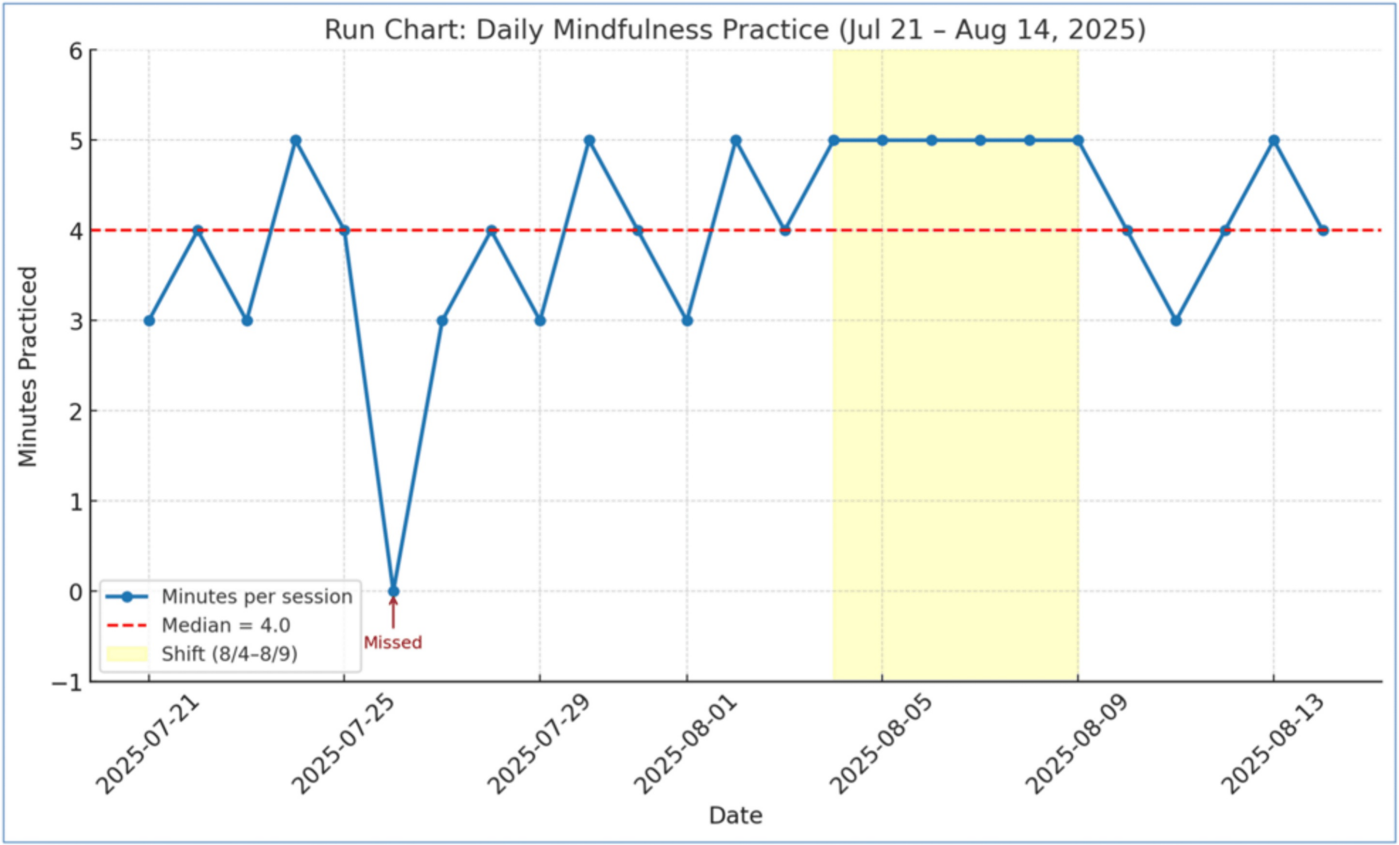
Daily duration of Daily Micro Moment (DMM) sessions (run chart) Daily duration of Daily Micro Moment (DMM) sessions over the three-week pilot period. The median session length was four minutes (red dashed line), with 92 % of sessions within the intended three to five-minute range. This run chart demonstrates strong implementation fidelity and consistent integration of brief restorative pauses into the workflow

These findings confirm that DMMs were feasible, sustainable, and compatible with real-world clinical and academic workloads. Adherence and fidelity data illustrated consistent engagement, meeting the predefined goal threshold of ≥85% (Figure 1). Session duration remained stable throughout the project, with a median of four minutes and 92% of sessions within the three to five-minute range (Figure 2). Together, these results demonstrate strong implementation fidelity-regular participation without time drift-supporting the feasibility of embedding brief, intentional pauses within routine workflow.

Reflections

Reflections recorded in the structured logbook showed a clear shift in perceived burden and integration of DMMs over time. During the first week, the practices occasionally felt like an additional task (burden ≤2 on a 1-5 scale). By the second week, DMMs became routine-short, intentional pauses that blended naturally into the rhythm of work and study. Weekly entries described these moments as providing calm and re-centering, particularly during transitions between academic assignments and weekend inpatient duties. The physician noted smoother role transitions, reduced stress reactivity, and greater focus after each brief pause. These qualitative impressions paralleled quantitative trends: perceived-stress ratings declined from 8 → 6 → 5, and the Mini-Z burnout score improved by one level, reinforcing the impression that DMMs enhanced focus and presence without adding workload.

Balancing measures

Balancing measures were tracked weekly to ensure that DMMs did not interfere with academic or clinical performance. Perceived burden remained low (median ≤2 on a 1-5 scale) and declined slightly over time. Time displacement from core responsibilities was negligible-less than 10 minutes per week-with no missed coursework deadlines, lapses in participation, or delays in documentation. Qualitative reflections supported these findings: rather than creating disruption, DMMs were perceived as improving concentration and reducing feelings of overwhelm during peak workload periods. These results confirm that the intervention was non-disruptive and compatible with ongoing professional responsibilities during the feasibility phase.

Early outcomes

The Mini-Z single-item burnout score (0-4 scale) was used to assess self-reported burnout at baseline and after the three-week intervention. A summary of outcome, process, and balancing measures is presented in Table 3. The burnout score decreased by one level, meeting the predefined project aim. Weekly perceived-stress ratings (0-10 scale) also showed a consistent downward trend from 8 → 6 → 5.

**Table 3:** Summary of outcome, process, and balancing measures used in the Daily Micro Moments (DMM) feasibility pilot Table 3 summarizes the outcome, process, and balancing measures that guided evaluation of the Daily Micro Moments (DMM) pilot. Results demonstrate improved well-being scores and high adherence without disruption of academic or clinical responsibilities

Measure type	Metric	Tool/scale	Frequency/timepoints	Result/trend
Outcome	Mini-Z single-item burnout score (0–4)	Validated survey item	Baseline/week 3	↓ by 1 level (meeting project aim)
Secondary outcome	Perceived-stress rating (0–10)	Self-rating	Weekly	8 → 6 → 5 (decreasing trend)
Process 1	Adherence (% days completed)	Daily logbook	Daily/weekly	20 of 25 days (80 %)
Process 2	Duration (minutes per session)	Logbook	Daily	92 % within 3–5 min target
Process 3	Timing reliability (% within chosen window)	Logbook	Daily	≈ 80 % within the designated window
Balancing 1	Perceived burden (1–5 scale)	Weekly rating	Weekly	≤2 and declining
Balancing 2	Time displacement (min/week)	Self-estimate	Weekly	<10 min per week; no missed tasks

**Figure 3:**
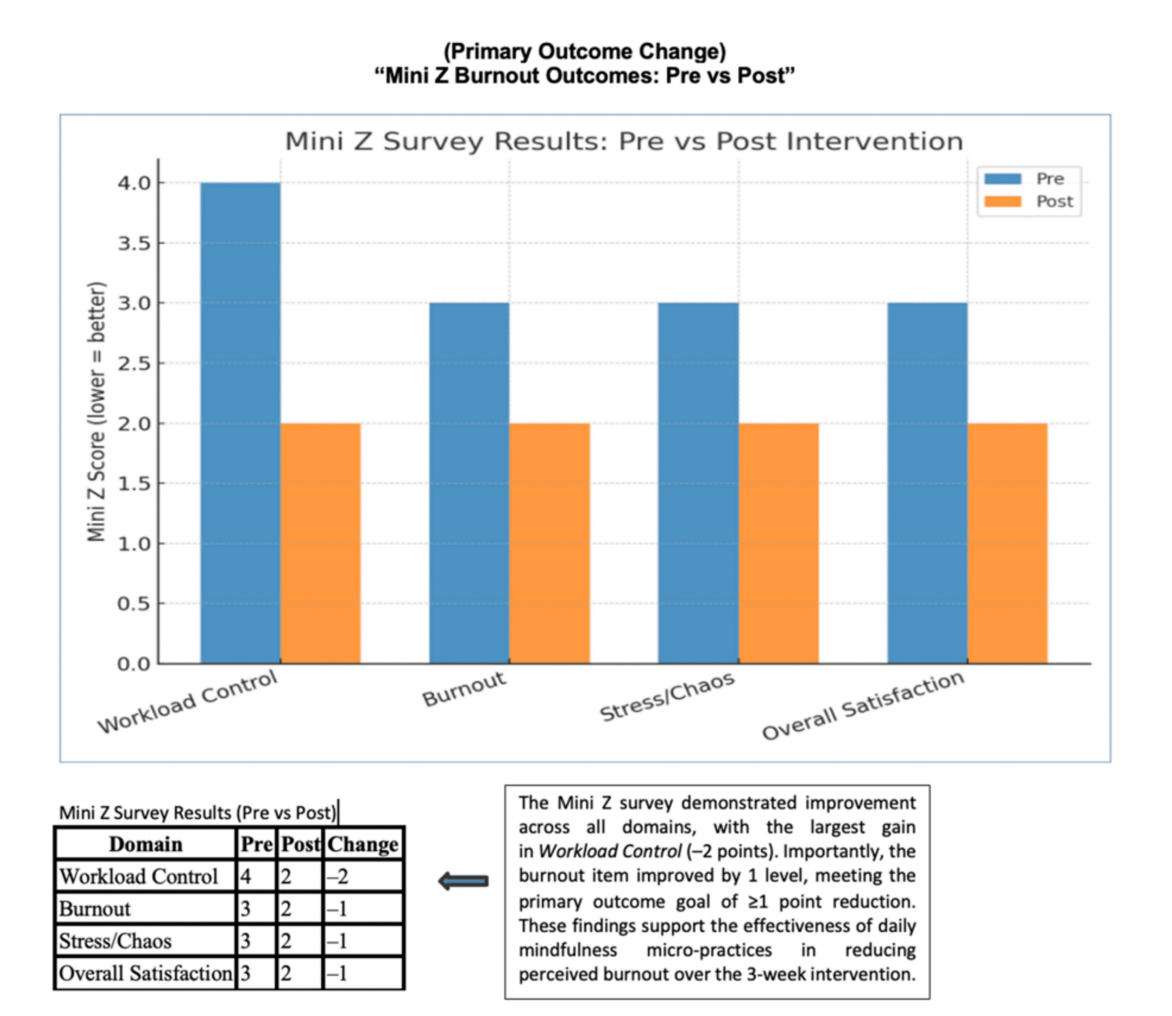
Mini-Z burnout outcomes: pre- vs. post intervention Mini-Z survey results before and after the three-week DMM intervention across four domains: workload control, burnout, stress/chaos, and overall satisfaction. Scores improved across all domains, with the largest gain in workload control (-2 points) and a one-level reduction in burnout, meeting the predefined project aim. These results highlight the potential of Daily Micro Moments (DMM) to improve perceived well-being and workflow control

Quantitative Mini-Z data demonstrated improvement across all measured domains following implementation of DMMs. Figure 3 summarizes pre- and post-intervention scores, showing a one-level reduction in burnout, stress, and overall satisfaction, and a 2-point improvement in perceived workload control. These findings align with qualitative reflections of reduced reactivity, smoother transitions, and enhanced focus, reinforcing that brief, structured pauses can meaningfully improve perceived well-being during the workday.

Across all Mini-Z domains, the greatest improvement was observed in perceived workload control (-2 points), underscoring the feasibility of integrating DMMs into existing clinical and academic workflows. The physician described less reactivity to stressors, smoother transitions between roles, and heightened awareness of the restorative value of short, intentional pauses.

Together, these findings indicate that DMMs may enhance focus, presence, and resilience without requiring additional time allocation. The pilot’s feasibility outcomes suggest potential for broader application among clinicians, residents, and students managing similarly demanding dual-role workloads.

## Discussion

This pilot demonstrates that incorporating DMMs into the routine of a busy physician is both feasible and beneficial. Beyond feasibility, the experience revealed practical insights into accountability, flow, and resilience that may inform future wellness frameworks. Although tested in the context of dual academic and clinical responsibilities, these findings may generalize to physicians, trainees, and students managing varied workload demands. A central observation was the role of accountability. The structured logbook transformed simple pauses into intentional acts, reframing wellness as a series of achievable daily goals rather than abstract ideals. This approach parallels established quality-improvement principles, where feedback and measurement sustain engagement [5].

Equally notable was the subjective experience of DMMs. Even brief pauses-such as mindful breathing or reflective journaling-functioned as “resets,” interrupting stress accumulation and promoting smoother transitions between roles. Rather than competing with clinical duties, these micro-practices appeared to enhance workflow and attention. This directly addresses a recurring barrier in clinician-wellness efforts: the perception that self-care detracts from professional performance [3,4]. The evolving sense of burden over time offers additional insight. Initially perceived as an “extra step,” DMMs became progressively integrated and automatic. By the second week, participants described them as “mini-resets” that improved focus and efficiency. This trajectory mirrors habit-formation research showing that repeated behaviors become increasingly effortless and self-reinforcing over time [6].

Although this pilot relied on self-report and qualitative reflection, the logbook data indicated meaningful improvement. The simple act of documentation provided a visible record of progress and reinforced adherence. Future studies could expand upon these findings using validated instruments to more precisely measure burnout and resilience outcomes. In sum, this project illustrates that resilience can be cultivated across stages of medical training and practice when interventions are brief, flexible, and embedded within daily workflows. By positioning wellness as integral to, rather than separate from, professional life, DMMs represent a pragmatic and sustainable model for mitigating stress and burnout in both learning and practice environments.

## Conclusions

This pilot highlights the potential of DMMs as a simple and feasible framework for promoting well-being in demanding medical environments. Even brief, intentional pauses fostered a sense of control, eased feelings of overwhelm, and restored connection with one’s inner self. For the physician in this report, DMMs served as moments of calm and reward-small resets that sustained focus and resilience throughout the day. Given the persistently high stress levels faced by physicians, trainees, and students, integrating wellness practices into natural transitions holds particular promise. While limited in scope, this pilot suggests that DMMs provide an accessible, low-burden approach to cultivating resilience without requiring additional scheduled time. Future studies should evaluate DMMs in larger, diverse cohorts using validated instruments to determine their broader impact and scalability.
